# Analysis of Volleyball Video Intelligent Description Technology Based on Computer Memory Network and Attention Mechanism

**DOI:** 10.1155/2021/7976888

**Published:** 2021-12-28

**Authors:** Zhongzi Zhang

**Affiliations:** Division of Sports Science and Physical Education, Northeastern University at Qinhuangdao, Qinhuangdao 066004, Hebei Province, China

## Abstract

There are some problems in the process of video intelligent description and analysis of volleyball, such as poor effective information extraction rate and poor dynamic tracking effect. Based on this, combined with long-term and short-term memory network and attention mechanism, this paper designs an intelligent description model of volleyball video based on deep learning algorithm and studies how to improve the extraction rate of volleyball video information through intelligent detection hardware and image recognition technology. This paper first introduces the application of image recognition technology and deep learning algorithm in the intelligent description of volleyball video, then designs the volleyball video and image recognition model based on deep learning algorithm according to the requirements of volleyball video intelligent description, and selects three correlation factors related to the impact indicators of volleyball skills. This study selects three characteristic parameters associated with volleyball video analysis indexes, namely, take-off, bounce, and hand movement, combined with image sensing hardware assisted sensor network to realize real-time monitoring of action state in volleyball video analysis system. The experimental results show that, compared with the current mainstream sports video intelligent analysis and image recognition methods with data analysis as the core, the intelligent volleyball sports video intelligent description and image recognition system based on the integration of deep learning algorithm and sensor hardware assistance has the advantages of good detection effect, high data effectiveness, low cost, and high efficiency of volleyball sports video analysis. It can effectively improve the efficiency of volleyball video intelligent description.

## 1. Introduction

In recent years, China's sports industry has entered a new period of rapid development. Volleyball plays an important role in sports [1]. Intelligent sports video analysis system based on intelligent algorithm processing is an essential part of modern education system and an important part of promoting the diversification of volleyball. It includes a video acquisition module for collecting teaching video, a video processing module for processing teaching video, and a video output module for outputting detection results. The video acquisition module includes a plurality of indoor cameras and an image preprocessing module connected with the cameras. Through teaching pixel level modeling and processing, complete and high-quality teaching detection lays a good foundation for moving target recognition and behavior judgment [2]. Sports reflect the development level of national education, science, and technology from the side [3]. However, in the process of competition, China's current sports often only pay attention to the athletes' mastery of sports skills and actual combat but ignore how to effectively realize more efficient volleyball video detection with the help of current technologically mature sensing hardware and intelligent algorithms [4]. In the process of describing volleyball video events, due to the problem of video information extraction effect, commentators often only pay attention to the skill mastery needs of sports but ignore the “repeated” detection of volleyball video, which violates the development concept of “intelligent volleyball video and intelligent description” [5]. The deep learning algorithm in image recognition technology can realize the deep mining of limited data information in video or image in a variety of fields and has the functions of high intelligence, good stability, and wide application range [6]. In this context, combined with long-term and short-term memory network and attention mechanism, this paper studies the intelligent description model of volleyball video based on deep learning algorithm.

In view of the low degree of effective information in information description and feature extraction of volleyball video, data mining analysis method is used first. Combined with long-term and short-term memory network, attention mechanism, and image recognition technology, this paper studies the intelligent description model of volleyball video based on deep learning algorithm. This paper is divided into four parts. The first part briefly describes the research background and research framework. The second part summarizes and analyzes the research status of intelligent description and information extraction of volleyball video. In the third part, combined with long-term and short-term memory network, attention mechanism, and image recognition technology, three correlation feature parameters related to the impact index of volleyball video intelligent description quality are selected, and a volleyball video intelligent description model based on correlation factor feature parameters and deep learning algorithm is proposed. The fourth part designs relevant experiments to verify the feasibility of the intelligent model proposed in this study and makes an objective quantitative evaluation of the experimental results through the quantitative evaluation method to draw a conclusion.

Compared with the current mainstream volleyball video intelligent description model (mainly greedy iterative algorithm or key feature intelligent extraction data analysis algorithm), the innovation of this paper is to propose an improved deep learning algorithm combined with long-term and short-term memory network, attention mechanism, and image recognition technology. It can make full use of the information of volleyball video intelligent description technology to realize the overall capture and information extraction of key actions in volleyball. By combining image recognition technology, it can realize the extraction, analysis, and correlation coincidence of target information, complete the coincidence analysis of influencing factors with quantitative indicators, and realize the key information analysis of sports video intelligent description.

## 2. State of the Art

Up to now, most volleyball video intelligent analysis and description in China have problems of weak foundation and low intelligence, especially in the construction of sports video intelligent description and detection system [7]. Moreover, in the previous research on volleyball video analysis system abroad, the recognition effect of the method used is poor [8]. Aiming at the problem of low training efficiency in volleyball, Qi et al., combined with the long-term and short-term memory network and video feature capture method, put forward a real-time feedback volleyball teaching analysis model, which can effectively improve the action error correction effect of college students in the process of volleyball. However, a large number of data samples are needed for strategy learning to achieve a certain degree of self-adaptation [9]. Bisagno et al. improved the process of volleyball video analysis in combination with the existing pyramid algorithm and proposed a pyramid algorithm combined with the idea of neural network. Using the data judgment dimension of volleyball video in the process of information extraction, they realized the information deep learning and high accuracy recognition of volleyball action, but its recognition efficiency was slow [10]. Benelguemar et al. put forward a fast recognition method by analyzing the uniqueness of volleyball movements and according to the actions with obvious distinction in the display process of volleyball, but the recognition types of this method are less [11]. In order to improve the recognition speed of actions in volleyball video, according to the different information of different athletes in the performance of the same type of volleyball action, combined with image processing technology, and aiming at the same kind of volleyball action, Zhou et al. put forward a volleyball video analysis method which can be fast and accurate among volleyball players. However, its application range is very limited [12]. Nam et al. found that the recognition effect of current volleyball movement is related to the surrounding environmental information, so they proposed a volleyball video analysis method to eliminate the influence of environmental noise, but there are problems of low recognition accuracy and poor stability [13]. Fern et al. found that, in the process of analyzing relevant actions in volleyball sports events, key video analysis nodes have a great impact on the overall recognition effect accuracy [14]. Gui et al. combined with pyramid algorithm proposed a fast recognition model for key sports actions in volleyball video. The experimental results show that this recognition method has higher recognition efficiency and recognition speed than the conventional volleyball video analysis method, but it is impossible to make efficient use of the action data in volleyball video. It is also impossible to conduct in-depth analysis at the data level [15]. Zhou et al. put forward a new “point-to-point” volleyball video intelligent analysis model by studying and analyzing the performance stability and action similarity of athletes in the process of performing volleyball [16]. MTB et al. verified the effectiveness of the deep learning recognition model in the process of volleyball video analysis through practical experiments on several volleyball athletes [17].

To sum up, it can be seen that the current volleyball video analysis system and the traditional image recognition model generally have the problems of poor recognition effect, low recognition accuracy, poor stability, and low data utilization in volleyball video analysis [18, 19]. On the other hand, in the existing volleyball video analysis system, the vast majority of volleyball video intelligent recognition methods can only recognize a single volleyball video information and can not distinguish volleyball sports with obvious differences, so they do not have intelligent characteristics [20–22]. And the utilization rate and data mining effect of the obtained volleyball movement data information in the recognition process are also very poor [23, 24]. Therefore, it is of great value to carry out the research on volleyball video intelligent description technology combining long-term and short-term memory network and attention mechanism.

## 3. Methodology

### 3.1. Application of Image Recognition Technology and Deep Learning Algorithm in Volleyball Video Analysis Model

**Figure 1:**
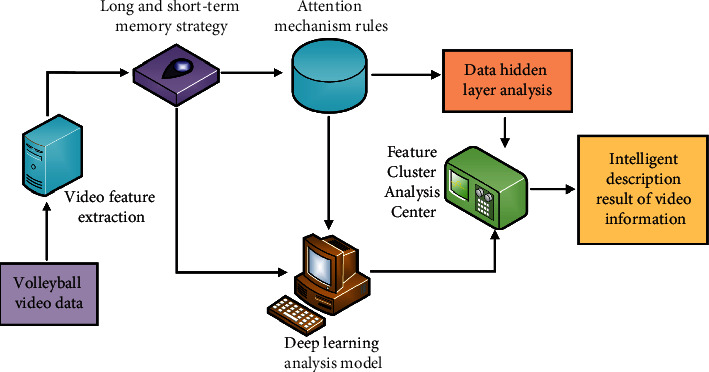
Data processing process of deep learning analysis model combined with long- and short-term memory and attention mechanism.

Image recognition technology is an important field of artificial intelligence. It refers to the technology of object recognition of images to identify targets and objects of different modes [25]. If the action in volleyball video needs to be extracted by intelligent analysis and description, it needs to be realized by intelligent algorithm in image recognition technology [26]. With the development of artificial intelligence technology, in terms of data processing of video information, image recognition technology based on deep learning algorithm has become a research hotspot and development trend in the fields of video and image information mining and data association analysis [27, 28]. What video intelligent description technology needs to solve is to divide the target data set in the image or video group into multiple groups according to a certain correlation law. Generally speaking, it is a process in which the data is classified into one group according to the similarity or contribution of the target task, and the difference of relevant data information or contribution is classified into different groups. In this paper, in the information processing of volleyball related videos and images, the observation object is volleyball data, and the observation index is the management degree between and within volleyball video data. The databases learned by the deep learning algorithm are volleyball match videos, physical education teaching videos, other volleyball videos, etc., published on the Internet. The total amount of data is obtained by the algorithm through simulation based on Internet video. Through in-depth learning, the obtained database is output directly. The results show that the total amount of data is more than 10 million times. The learning rules include input layer, hidden layer, and output layer. The input layer is volleyball video, and the hidden layer is the process of performing mathematical calculation on the input data. The calculation method is mainly an adaptive iterative cyclic calculation mode based on successive error reduction, and the output layer is the target data result (that is, the intelligent description of volleyball video). The data processing process of the deep learning analysis model combining long-term and short-term memory and attention mechanism is shown in Figure 1.

### 3.2. Construction of Volleyball Video Intelligent Description Model Based on Long-Term and Short-Term Memory Network and Deep Learning Algorithm

The volleyball video recognition algorithm based on deep learning is used to analyze and process the image. The different motion trajectories of volleyball in different motion scenes are photographed, and several photos are input into the system as training sets for model training. Finally, the model is tested. The above types of photos different from the sample set are used as the test set to test the fitting degree of the model. According to the test results, the model can be continuously optimized to improve the recognition accuracy and system performance.

The combination of attention mechanism and long-term and short-term memory network with attention mechanism can improve the accuracy of quality rating. This method overcomes the disadvantage of slow text model training and adapts to the end-to-end model analysis of data features. Through the methods of recognition, representation, and feature weighting, the long text is accurately classified by quality rating.

**Figure 2:**
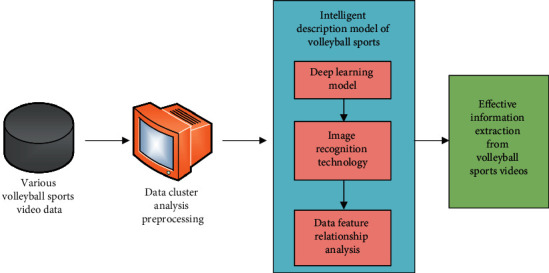
The data analysis process of the volleyball video intelligent description model.

This volleyball video analysis model based on deep learning algorithm combines long-term and short-term memory network and attention mechanism to simplify the processing of complex data in volleyball video information. Therefore, the intelligent description model needs to check many factors of multidata, so as to judge whether there are closely related factors in a variety of data. In this way, it can not only use the comprehensive average index of these closely related factors or one of them to represent a variety of such factors, but also make these multidata to be processed. The information carried by video and image is not seriously distorted. The data analysis process of its volleyball video intelligent description model is shown in Figure 2.

Firstly, we assume that there are *n* data objects to be processed (athlete feature information in video and image in the process of volleyball), which are called implementation objects; then the position sequence expression of volleyball in volleyball video in three-dimensional space is

(1) $$\begin{array}{c} X_{1}=\left(x_{1}\left(1\right),x_{1}\left(2\right),\ldots ,x_{1}\left(n\right)\right),\\ X_{2}=\left(x_{2}\left(1\right),x_{2}\left(2\right),\ldots ,x_{2}\left(n\right)\right),\\ Y_{1}=\left(y_{1}\left(1\right),y_{1}\left(2\right),\ldots ,y_{1}\left(n\right)\right),\\ Y_{2}=\left(y_{2}\left(1\right),y_{2}\left(2\right),\ldots ,y_{2}\left(n\right)\right),\\ Z_{1}=\left(z_{1}\left(1\right),z_{1}\left(2\right),\ldots ,z_{1}\left(n\right)\right),\\ Z_{2}=\left(z_{2}\left(1\right),z_{2}\left(2\right),\ldots ,z_{2}\left(n\right)\right), \end{array}$$

where *x*, *y*, *z* are the spatial trajectory coordinate information of volleyball, *X*, *Y*, *Z* are the motion position of volleyball in three-dimensional space, and *n* is the total amount of different volleyball video feature points.

After combining the long-term and short-term memory variable function *K*(*x*, *y*, *z*) and the attention mechanism rule function *L*(*x*, *y*, *z*), the corresponding volleyball spatial position expression is

(2) $$\begin{array}{c} X_{1}^{\prime }=\frac{\left(x_{1}\left(1\right),x_{1}\left(2\right),\ldots ,x_{1}\left(n\right)\right)}{K\left(x,y,z\right)+L\left(x,y,z\right)},\\ X_{2}^{\prime }=\frac{\left(x_{2}\left(1\right),x_{2}\left(2\right),\ldots ,x_{2}\left(n\right)\right)}{K\left(x,y,z\right)+2L\left(x,y,z\right)},\\ Y_{1}^{\prime }=\frac{\left(y_{1}\left(1\right),y_{1}\left(2\right),\ldots ,y_{1}\left(n\right)\right)}{K\left(x,y,z\right)+nL\left(x,y,z\right)},\\ Y_{2}^{\prime }=\frac{\left(y_{2}\left(1\right),y_{2}\left(2\right),\ldots ,y_{2}\left(n\right)\right)}{K\left(x,y,z\right)+n^{2}L\left(x,y,z\right)},\\ Z_{1}^{\prime }=\frac{\left(z_{1}\left(1\right),z_{1}\left(2\right),\ldots ,z_{1}\left(n\right)\right)}{n^{2}K\left(x,y,z\right)+\left(n+1\right)L\left(x,y,z\right)},\\ Z_{2}^{\prime }=\frac{\left(z_{2}\left(1\right),z_{2}\left(2\right),\ldots ,z_{2}\left(n\right)\right)}{{\left(n+1\right)}^{2}K\left(x,y,z\right)+\left(n+2\right)L\left(x,y,z\right)}. \end{array}$$

The expressions of long-term and short-term memory variable function *K*(*x*, *y*, *z*) and attention mechanism rule function *L*(*x*, *y*, *z*) are, respectively,

(3) $$\begin{array}{c} K\left(x,y,z\right)=\frac{yx^{2}+xy^{2}+xyz^{2}}{x+y+z},\\ L\left(x,y,z\right)=\frac{zy^{2}x^{3}+xy^{3}+x^{-1}y^{-1}z^{3}}{x^{2}+y^{2}+z^{-1}}. \end{array}$$

Among them, *X*, *Y*, and *Z* are the motion position functions of volleyball in three-dimensional space, *n* is the total amount of video feature points of different volleyball sports, and *x*, *y*, *z* are the spatial trajectory coordinate information of volleyball sports. When the set signal is different from the signal in the volleyball video, combined with the analysis of the long-term and short-term memory network function, the deviation proportion, integral, and differential can be combined to form the control quantity, and the integral expression *P*(*t*) in the volleyball video intelligent analysis model is obtained as follows:

(4) $$\begin{array}{c} P\left(t\right)=k_{p}\left[e\left(t\right)+\frac{1}{T_{1}}\int_{0}^{t}e\left(t\right)\text{d}t+{T\;}_{D}\frac{de\left(t\right)}{dt}\right], \end{array}$$

where *e*(*t*) represents deviation, *k*_*p*_ represents control quantity, and *T* represents time constant.

### 3.3. Data Processing Process of Volleyball Video Intelligent Description System Based on Deep Learning

**Figure 3:**
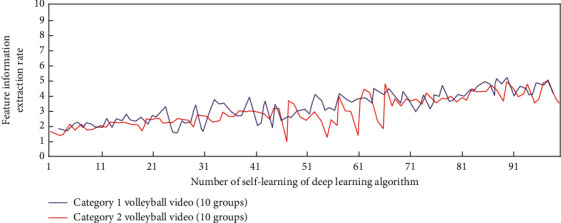
The simulation analysis process of feature information extraction in volleyball video.

**Figure 4:**
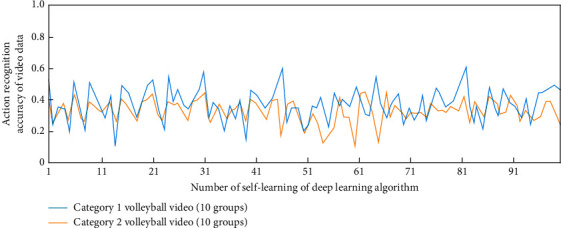
The extraction and simulation analysis process of action recognition in volleyball sports videos.

In this study, once the intelligent description decision of sports video is determined, the depth learning equation required in volleyball video can be determined. In order to study the influence of deep learning times on video information feature extraction, without combining long-term and short-term memory and attention mechanism, the simulation results of feature information extraction and action recognition in two categories (10 groups each) of volleyball video under different deep learning times are shown in Figures 3 and 4.

It can be seen from Figures 3 and 4 that when the long-term and short-term memory and attention mechanism are not combined, under different deep learning times, the deep learning algorithm has a gradually increasing trend in extracting the feature information from the volleyball video of two categories (10 groups in each group), but when the self-learning times are greater than 91, the maximum value of the feature information extraction factor is only about 5. In terms of action recognition, they are all in the recognition accuracy range of 0–0.6. This is because in order to determine the best volleyball video intelligent description and detection scheme, there is no combination of long-term and short-term memory and attention mechanism, which leads to the need to operate the array in reverse in the process of data learning; that is, the decision is determined according to the correlation degree of the relationship between the independent basic video intelligent description states of two adjacent segments. The sports skill video intelligent description model under the deep learning algorithm will be monitored in real time by the sensor hardware sensor network and then recorded by the sports basic video intelligent description system. The generated data will be optimally processed and analyzed by the deep learning algorithm, and these data information will be converted into binary numbers. Then the data is processed by computer network system to realize the analysis of volleyball video data.

**Figure 5:**
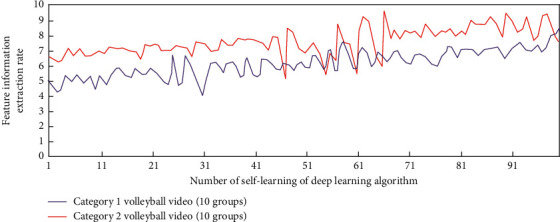
After the introduction of key factors, the simulation analysis process of the feature information extraction from the volleyball sports video.

**Figure 6:**
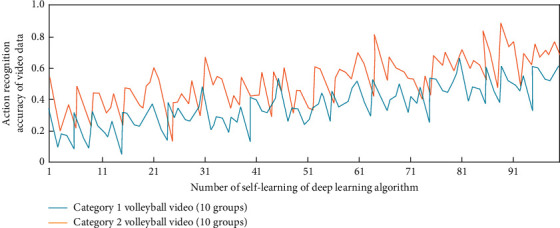
The process of simulation analysis of the accuracy of action recognition in volleyball sports videos after introducing key factors.

In this intelligent description model of volleyball video based on deep learning algorithm and sensor hardware assisted fusion, combined with the key factors of long-term and short-term memory variables and attention mechanism rules, the simulation results of feature information extraction and action recognition in volleyball video under different deep learning times are shown in Figures 5 and 6.

As can be seen from Figures 5 and 6, after combining the long-term and short-term memory variables and the key factors of attention mechanism rules, the corresponding feature information extraction and action recognition efficiency will be significantly improved with the increase of the amount of self-learning. This is because, in the process of finding the boundary conditions of sports video analysis system based on sensor hardware assisted fusion, the deep learning model can obtain one or a series of state transition reference values through multiple cycle learning steps. Generally, through the combination of long-term and short-term memory network and attention mechanism, the relevant data of volleyball in the process of sports rotation can be deeply mined and analyzed, and multiple comparative analysis can be carried out according to the volleyball action law in the deep learning database. After data processing and operation, the optimal probability analysis can be carried out.

## 4. Result Analysis and Discussion

### 4.1. Verification and Experimental Design of Volleyball Video Intelligent Description System

**Figure 7:**
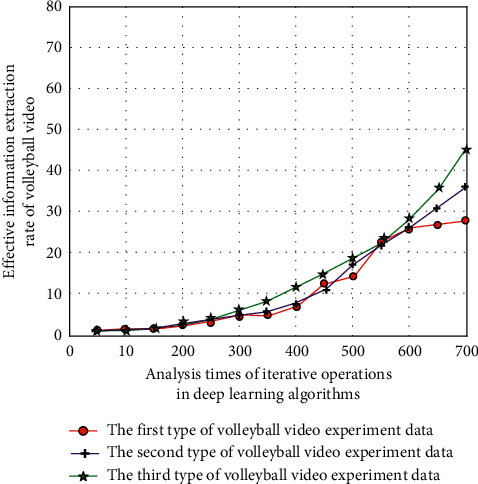
Effective information extraction rate without combining long and short-term memory networks and attention mechanisms.

When setting the confirmatory experiment, the database of in-depth learning is the volleyball event video, physical education teaching video, and other volleyball videos published on the Internet. The total amount of data is more than 10 million times. The learning rules include input layer, hidden layer, and output layer, in which the input layer is volleyball video. The hidden layer is to perform mathematical calculation on the input data. Its calculation method is mainly an adaptive iterative cyclic calculation mode based on successive error reduction, and the output layer is the target data result (i.e., intelligent description of volleyball video). When evaluating the application of deep rest learning algorithm in volleyball video intelligent description system, it needs to be evaluated from many aspects. Therefore, this study selects three associated characteristic parameters related to volleyball video analysis indicators, namely, starting, bouncing, and hand movement, combined with image sensing hardware assisted sensor network to realize the real-time monitoring of action status in volleyball video analysis system, and then transmits these monitored information data to the terminal database of detection system. Through the processing of dynamic algorithm and grey correlation entropy analysis method, the effective data information is extracted. The large data information (obtained by image sensing hardware assisted sensor network) generated in the system of the fusion of volleyball video intelligent description technology based on video intelligent description algorithm and sensing hardware assistance is obtained, and the corresponding quantitative evaluation results are obtained. In the experiment, for three types of data (according to the change degree of volleyball movement, it can be divided into three types of data, and two groups of volleyball video are selected for each type of data), when the long-term and short-term memory network and attention mechanism are not introduced, the preliminary experimental results on the effective information extraction rate in volleyball video are shown in Figure 7 with the increase of the number of iterative operations.

**Figure 8:**
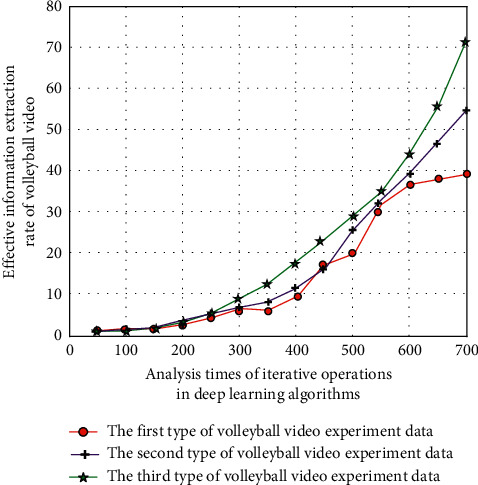
Effective information extraction rate when combined with long- and short-term memory network and attention mechanism.

After the introduction of long-term and short-term memory network and attention mechanism, the preliminary experimental results of effective information extraction rate in volleyball video are shown in Figure 8.

As can be seen from Figures 7 and 8, after introducing the long-term and short-term memory network and attention mechanism, under the deep learning algorithm, with the increase of the number of iterative operations, the corresponding volleyball video information extraction rate has been greatly improved. This is because the intelligent description system model of college volleyball video based on deep learning algorithm has the intelligent characteristics of self-learning and in-depth analysis, and these characteristics can realize multiple iterative analysis of effective information in volleyball video according to the long-term and short-term memory network and attention mechanism rules, so as to reduce the loss rate of effective information.

### 4.2. Experimental Results and Feedback Analysis of Intelligent Sports Video Analysis System

With the three types of data in the experiment (according to the change degree of volleyball movement, it can be divided into three types of data, and each type of data selects two groups of volleyball video), the analysis results based on the depth learning algorithm are shown in Table 1, where S1 represents the first volleyball video and D1 represents the first description results and (Sx, Dy) represents the quantitative description result of the *x* volleyball video at the *y* time.

**Figure 9:**
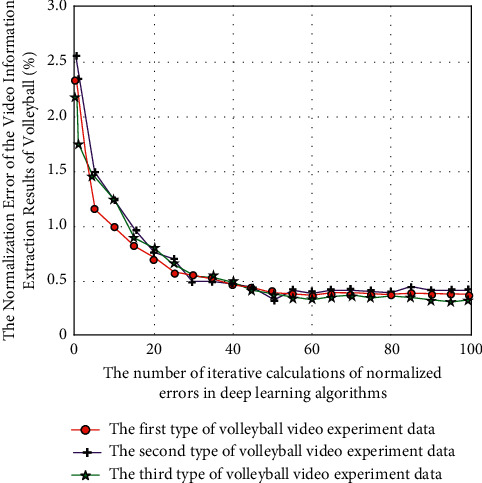
The normalized quantitative evaluation and analysis results of the error degree of the experimental results.

The normalized quantitative evaluation and analysis results of the error degree of the experimental results are shown in Figure 9.

**Figure 10:**
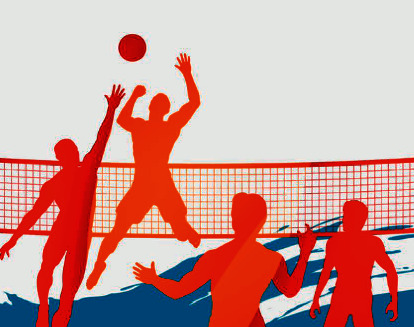
Silhouette illustration of volleyball.

It can be seen from Figure 9 that, among the three types of experimental data (six groups of experimental data), the change law of the corresponding quantitative evaluation results is similar. The error gradually decreases with the increase of the number of error iterative calculations. This is because although there are obvious differences in the feature analysis of the three types of experimental data, after the analysis of this volleyball video intelligent description model, different types of video data can be normalized, and the error can be reduced and stabilized in a certain range with the increase of the number of iterations, which also shows that the model proposed in this study has a certain migration application and scope of application and can analyze different types of volleyball video with high accuracy. On the other hand, the intelligent description model of volleyball video based on deep learning algorithm classifies information according to the individual differences between each player in 6 different groups of volleyball video and when they play volleyball in the video. Using image sensing hardware and sensor network fusion technology, the whole process of volleyball video dynamic monitoring is realized, and the dynamic process is monitored with data. The source data is transmitted to the system terminal through the Internet and then through the intelligent data depth mining technology and image recognition technology based on deep learning algorithm, so as to improve the accuracy and efficiency of intelligent description of volleyball video (see Figure 10).

## 5. Conclusion

With the popularity of Internet technology in the sports industry, how to use image recognition technology and intelligent analysis methods to intelligently describe and accurately identify volleyball video has become more and more important. On this basis, combined with long-term and short-term memory networks and attention mechanism, an intelligent description model of volleyball video based on deep learning algorithm is designed, and how to improve the extraction rate of volleyball video information through intelligent detection hardware and image recognition technology is studied. Compared with the current mainstream volleyball video intelligent description model (mainly greedy iterative algorithm or key feature intelligent extraction data analysis algorithm), the innovation of this paper is to propose an improved deep learning algorithm combined with long-term and short-term memory network, attention mechanism, and image recognition technology. It can make full use of the information in volleyball video intelligent description technology to realize the overall capture and information extraction of volleyball key movements. This paper first introduces the application of image recognition technology and deep learning algorithm in volleyball video intelligent description, then designs the volleyball video and image recognition model based on deep learning algorithm according to the requirements of volleyball video intelligent description, and selects three related factors related to the influence index of volleyball technology. The experimental results show that the intelligent volleyball video intelligent description and image recognition system based on the combination of deep learning algorithm and sensor hardware assistance has the advantages of good detection effect, high data efficiency, low cost, and high volleyball video analysis efficiency. It can effectively improve the efficiency of volleyball video intelligent description. However, the system only considers the information analysis of volleyball video data but does not consider the influence of volleyball players' sports habits on volleyball trajectory, so it can be further optimized from this aspect.

## Figures and Tables

**Table 1:** Analysis results of 6 sets of experimental data based on deep learning algorithms.

Ax/Vx	S1	S2	S3	S4	S5	S6
D1	0.13	0.48	0.13	0.23	0.22	0.43
D2	0.26	0.67	0.56	0.46	0.67	0.77
D3	0.33	0.89	0.79	0.75	0.95	0.88

## Data Availability

The experimental data used to support the findings of this study are available from the corresponding author upon request.
